# New Insights into Transcription Fidelity: Thermal Stability of Non-Canonical Structures in Template DNA Regulates Transcriptional Arrest, Pause, and Slippage

**DOI:** 10.1371/journal.pone.0090580

**Published:** 2014-03-03

**Authors:** Hisae Tateishi-Karimata, Noburu Isono, Naoki Sugimoto

**Affiliations:** 1 Frontier Institute for Biomolecular Engineering Research (FIBER), Konan University, Kobe, Japan; 2 Faculty of Frontiers of Innovative Research in Science and Technology (FIRST), Konan University, Kobe, Japan; Nazarbayev University, Kazakhstan

## Abstract

The thermal stability and topology of non-canonical structures of G-quadruplexes and hairpins in template DNA were investigated, and the effect of non-canonical structures on transcription fidelity was evaluated quantitatively. We designed ten template DNAs: A linear sequence that does not have significant higher-order structure, three sequences that form hairpin structures, and six sequences that form G-quadruplex structures with different stabilities. Templates with non-canonical structures induced the production of an arrested, a slipped, and a full-length transcript, whereas the linear sequence produced only a full-length transcript. The efficiency of production for run-off transcripts (full-length and slipped transcripts) from templates that formed the non-canonical structures was lower than that from the linear. G-quadruplex structures were more effective inhibitors of full-length product formation than were hairpin structure even when the stability of the G-quadruplex in an aqueous solution was the same as that of the hairpin. We considered that intra-polymerase conditions may differentially affect the stability of non-canonical structures. The values of transcription efficiencies of run-off or arrest transcripts were correlated with stabilities of non-canonical structures in the intra-polymerase condition mimicked by 20 wt% polyethylene glycol (PEG). Transcriptional arrest was induced when the stability of the G-quadruplex structure (−ΔG^o^_37_) in the presence of 20 wt% PEG was more than 8.2 kcal mol^−1^. Thus, values of stability in the presence of 20 wt% PEG are an important indicator of transcription perturbation. Our results further our understanding of the impact of template structure on the transcription process and may guide logical design of transcription-regulating drugs.

## Introduction

**Figure 1:**
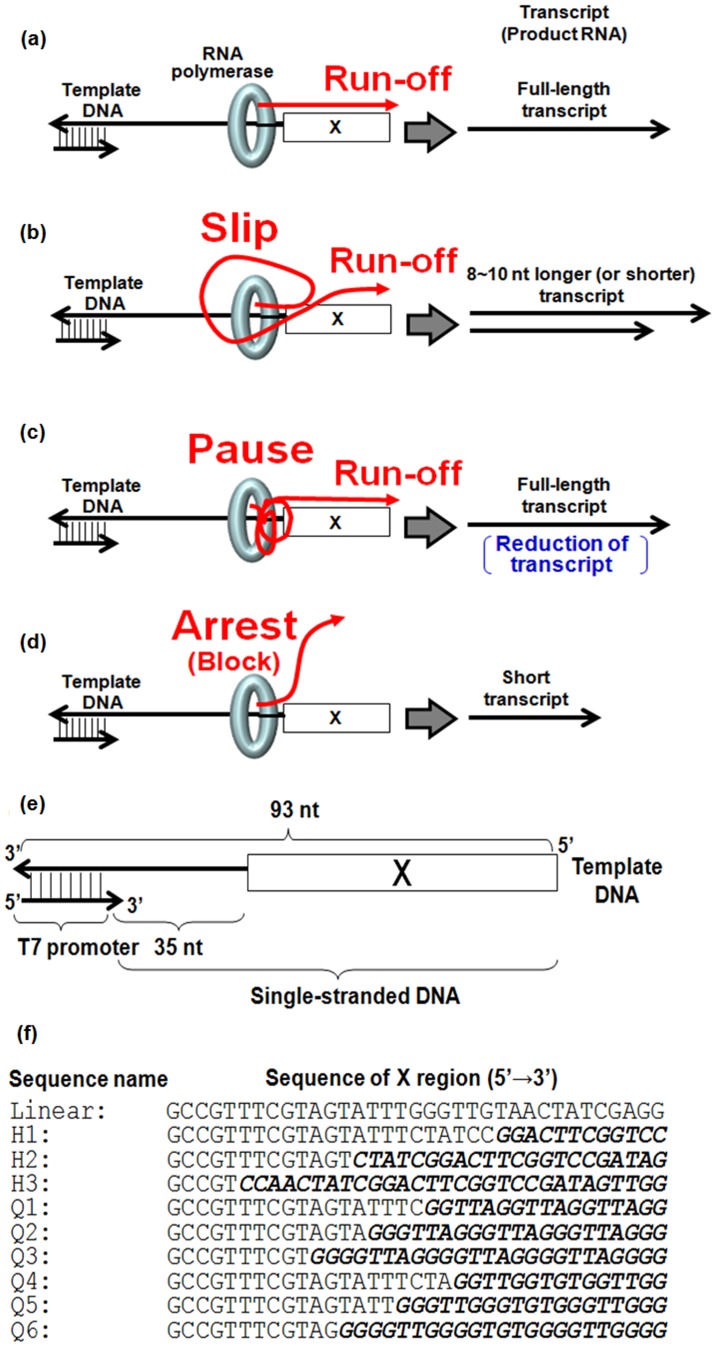
Effects on RNA polymerase elongation by structures in template DNA (a–d) and illustration of the template DNA (e, f). (a) An unstructured template, (b) a template with a slippage site, (c) a template with a pause site, and (d) a template with an arrest site. (e) The region denoted by the box marked with an X contains the sequence designed to form a random coil or non-canonical structure. (f) Sequence names and sequences of X regions. Sequences expected to form non-canonical structures are highlighted by italic and bold.

Transcription is the first step in gene expression; it is highly regulated during both initiation and elongation.[1], [2] Although fidelity of transcription elongation is critical for maintaining the accurate flow of genetic information, transcription elongation in cells and *in vitro* (Figure 1a) can be interrupted by certain sequences or structures.[3], [4] For example, the RNA polymerase may slip back or forward on a template DNA during RNA synthesis at “slippage” sites such as the homopolymeric tract in human *amyloid precursor protein* gene.[2], [5] In the *amyloid precursor protein* gene, the slippage results in a transcript 8- to 10-nt shorter or longer than the expected transcript, changing the coding capacity of mRNA (Figure 1b).[4], [5], [6] Moreover, the rate of transcription elongation is also dependent on sequence. The average rate of transcription elongation by RNA polymerase II on relatively unstructured DNA templates in the absence of any additional factors is ∼5 nucleotides s^−1^ under optimal conditions.[7], [8] At some template positions called “pause” sites such as the A-T-rich transactivation sequence in the HIV genome, the polymerase may halt for as long as 1 min before continuing transcription. The pause results in decreased mRNA production and therefore less protein production (Figure 1c). At other locations called “arrest” sites, which can be a damaged DNA residue or a an A or T stretch,[5], [6] a transcribing polymerase halts, and the 3′ end of the transcript is displaced from the active site of the polymerase and the short transcript is released (Figure 1d).[9] These arrested transcripts may misfold or result in translation of mutant protein. Like non-coding RNAs such as miRNAs and antisense RNAs,[10], [11], [12], [13], [14] these short transcripts may also have roles in regulation of gene expression. Transcription is important not only for cellular systems but is also in certain nano-materials such as logic devices.[15] If transcript mutations could be controlled, they might prove useful in medical, pharmaceutical, and materials sciences fields.

We have shown that non-canonical structures such as triplexes, G-quadruplexes, and cruciform structures are stabilized drastically under cell-like conditions induced by cosolutes,[16] although a duplex is destabilized under the same conditions.[16], [17] Recently, it has been reported that non-canonical G-quadruplex structures are present inside cells.[18] These results imply that non-canonical structures may play important roles in gene regulation. Additionally, we showed very recently that the formation of a G-quadruplex in an open reading frame (ORF) of mRNA suppresses its translation into protein.[19] It has been reported that quadruplex-forming sequences are enriched upstream and within gene promoters; structures formed in these regions may lead to transcription pausing via the inhibition of transcription initiation.[20] Moreover, hairpin structures formed by the nascent RNA may result in transcription pausing even if the hairpin does not form in template DNA.[21], [22] Recent reports have indicated that transcription is arrested prior to stable structures in the DNA template such as a Z-form duplex,[23] triplex,[24], [25] or G-quadruplex,[26], [27] but there have been no quantitative and systematic reports the effects of various non-canonical structures on arrest, slippage or pause of transcription from the viewpoint of stability of non-canonical structures at the energy level.

The phenomena of arrest, pause, and slippage are ubiquitous aspects of transcriptional regulation. Arrest, pause, and slippage sites may result from unstable hybridization between template DNA and nascent RNA or upon formation of a structure in the DNA that interrupts and perturbs elongation. Although formation for a DNA/RNA hybrid can result in transcriptional interruption,[28] the mechanism of transcription interruption due to non-canonical structures may differ, because non-canonical structures like G-quadruplex have very different structural features and stabilities than DNA/RNA hybrids. Here, we evaluated the quantitative effects of non-canonical structures formed in the template DNA on transcription catalysed by T7 RNA polymerase.

## Results

### Structure and stability of non-canonical structures

Hairpins (template region sequences of cruciform structure) and G-quadruplexes are known to form in template DNA. We designed and synthesized ten different template DNAs (Figures 1e, 1f and Table S1 in File S1) in order to evaluate the effect of formation of hairpins and G-quadruplexes with different thermal stabilities on transcription elongation. The control sequence (Linear) should not form significant structure. Sequences H1 to H3 and Q1 to Q6 were designed to form hairpins or G-quadruplexes, respectively, at a site 35 bases downstream from the T7 promoter region as shown in Figure 1e. The G-quadruplex-forming sequences are based on the human telomeric sequence and the thrombin DNA aptamer sequence and have different numbers of G-quartets (Figure 1f).[16] To confirm the formation of the non-canonical structures in the template DNAs, oligonucleotides containing only the non-canonical structure region (linear, h1 to h3, and q1 to q6) were synthesized (Table S2 in File S1). We measured circular dichroism (CD) spectra of these DNA oligonucleotides at 37°C (Figure S1a in File S1) and also analyzed each by native gel electrophoresis (Figure S1b in File S1). These analyses indicated hairpin formation by h1, h2, and h3, and G-quadruplex formation by oligonucleotides q1-q6 (Table 1 and Figure S1c in File S1). Moreover, formation of G-quadruplexes in the template DNAs for Q1-Q6 was confirmed by florescent analysis using protoporphyrin IX, which binds specifically to G-quadruplexes and produces fluorescence (Figure S2 in File S1).[19], [29]

**Table 1:** The stabilities of non-canonical structures designed to form in template DNA ^a^

Sequence^b^	Structure	*T*_m_ c (°C)	−Δ*G* ^o^_37_ (kcal mol^−1^)	Arrest^d^
H1 (h1)	hairpin	55.5	2.7±0.3	No
H2 (h2)	hairpin	74.5	7.2±0.4	No
H3 (h3)	hairpin	82.4	12.7±0.2	No
Q1 (q1)	antiparallel G-quadruplex	37.3	0.1±0.1	No
Q2 (q2)	(3+1) mixed G-quadruplex	62.9	3.7±0.2	No
Q3 (q3)	(3+1) mixed G-quadruplex	89.8	n.d.^e^	Yes
Q4 (q4)	antiparallel G-quadruplex	46.8	1.4±0.2	No
Q5 (q5)	parallel G-quadruplex	80.5	14.3±0.3	Yes
Q6 (q6)	antiparallel G-quadruplex	>95	n.d.^e^	Yes

a All experiments were carried out in a buffer containing 30 mM KCl, 40 mM Tris-HCl (pH 8.0), 8 mM MgCl_2_, and 2 mM spermidine.

b The sequences of template DNAs are shown in Figure 1b and Table S1 in File S1. The sequences designated by lower case letters contain only the non-canonical structure region (see Table S2 in File S1).

c The melting temperature was determined at a strand concentration of 2 µM.

d Arrest was defined as more than 4% production of arrested product RNA.

e The −Δ*G* ^o^_37_ value could not be determined because of very high stability.

**Figure 2:**
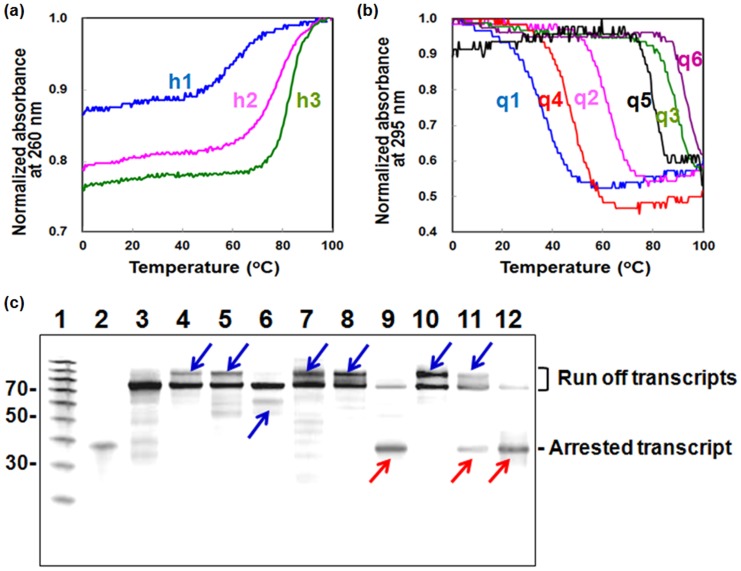
UV melting curves for oligonucleotides containing sequences in DNA templates (a, b) and effects of non-canonical structures in the template DNA on transcription (c). Normalized UV melting curves for 2 µM (a) h1 (blue), h2 (pink), and h3 (green), (b) q1 (blue), q2 (pink), q3 (green), q4 (red), q5 (black), and q6 (purple) in a buffer containing 30 mM KCl, 40 mM Tris-HCl (pH 8.0), 8 mM MgCl_2_, and 2 mM spermidine. The *T*_m_ values for h1, h2, h3, q1, q2, q3, q4, q5, and q6 were 55.5, 74.5, 82.4, 37.3, 62.9, 89.8, 46.8, 80.5 and >95°C. (c) Denaturing gel electrophoresis of products of transcription reactions carried out for 90 min at 37°C. Reaction mixtures contained 0.3 µM T7 polymerase and 1.5 µM DNA template in a buffer containing 30 mM KCl, 40 mM Tris-HCl (pH 8.0), 8 mM MgCl_2_, and 2 mM spermidine. Lane 1, size marker; lane 2, 35-nt RNA; lanes 3 to 12, transcription products for Linear, H1, H2, H3, Q1, Q2, Q3, Q4, Q5, and Q6 templates, respectively. Blue and red arrows indicate the slipped and arrested transcripts, respectively.

The thermodynamic parameters for the formation of hairpins and G-quadruplexes were obtained from their thermal melting curves by monitoring UV absorbance at 295 or 260 nm (Figure 2a).[16] The mid-points of the thermal melting transitions (*T*_m_ values) and thermodynamic parameters for the structure formation are given in Table 1 and Table S3 in File S1. The values of *T*_m_ and −Δ*G* ^o^37 (the stability at 37°C) for h1, h2, and h3 increased with increasing the stem length (4, 9, and 13 base pairs, respectively). The *T*_m_ values for G-quadruplexes based on the human telomeric q1, q2, and q3 were 37.3, 62.9, 89.8°C, respectively. Those for G-quadruplexes q4, q5 and q6 based on the thrombin aptamer sequence were 46.8, 80.5, and >95°C, respectively. In both cases, the *T*_m_ values increased with the number of G-quartet stacks. The values of −Δ*G* ^o^37 for the G-quadruplex-forming oligonucleotides also increased with increasing the number of G-quartets. The G-quadruplexes formed by q4, q5, and q6 were much more stable than those formed by q1, q2, and q3 due to differences in stacking interactions of loop regions (Figure S1c in File S1). These thermal analyses indicated that all the non-canonical structures should form in a template DNA at 37°C.

### Effect of non-canonical structures on transcription

T7 RNA polymerase transcription of the linear template under multi-turnover conditions was almost saturated at 90 min (data not shown). Inhibitory effects of non-canonical structures in the template DNA on transcription were estimated from the amount of transcript (product RNA) formed at this time point. Figure 2c shows the results of gel electrophoretic analysis of transcription carried out for 90 min at 37°C under multi-turnover conditions. RNA size was determined by analysis of samples of each reaction in parallel with size markers and a 35-nt RNA on a denaturing polyacrylamide gel (Figure 2c). When the template with no significant structure was used, the transcription proceeded to the end of the DNA template, resulting in the formation of a full-length transcript of 70 nt (Figure 2c, lane 3). To confirm that the Linear template produced mainly full-length transcript, we carried out additional experiments to quantitate formation of longer and shorter products from this template (Figure S3 in File S1 and Methods section). Interestingly, transcription of all template DNAs able to form non-canonical structures resulted in products in addition to the full-length transcript. The products of transcription of templates H1, H2, Q1, Q2, Q4, and Q5 yielded transcripts approximately 10-nt longer than the full-length transcript (Figure 2c, lanes 4, 5, 7, 8, 10, and 11). In contrast, transcripts from the reaction with H3, which formed the most stable hairpin, contained a minor product band that migrated at approximately 60 nt, about 10-nt shorter than the full-length transcript (Figure 2c, lanes 6). Previous studies indicate that elongating RNA polymerase can slip to produce transcripts longer and shorter by 8 to 10 nts. Thus, formation of non-canonical structures appears to induce slippage. The template DNAs Q3, Q5, and Q6 able to form the very stable G-quadruplexes (−Δ*G* ^o^37 values more than 14.3 kcal mol^−1^ in Table 1) induced production of a transcript of about 35 nt (Figure 2c, lanes 9, 11 and 12). This length suggests that T7 transcription was arrested at the G-quadruplex structure. Under the conditions used here, the hairpins induced slippage, whereas G-quadruplexes induced slippage and arrest, with extent of arrest depending on the G-quadruplex stability. We also carried out the transcription using template DNA in the presence of complementary strand (Figures S4a and S4b in File S1). Template DNAs of Q3, Q5, and Q6 induced production of the arrested transcript even in the presence of complementary DNA (Figure S4c in File S1). Thus, our model systems revealed the correlation between the stability of structures adopted by the template and the transcription efficiency.

### Amount of non-full length transcript depends on the stability of non-canonical structures

**Figure 3:**
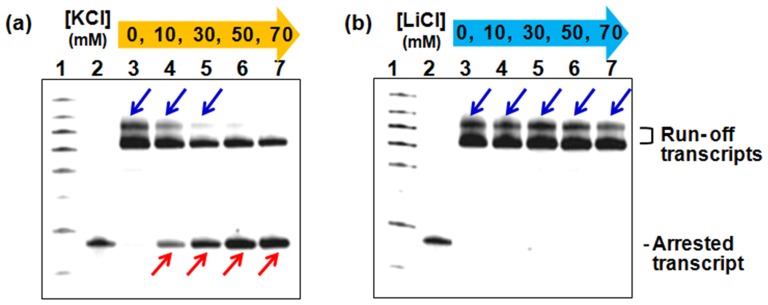
Effect of the G-quadruplex stability on the production of slipped and arrested transcripts. Denaturing gel electrophoresis of products of transcription reactions carried out for 90°C using Q5 template. Reaction mixtures contained 0.3 µM T7 polymerase and 1.5 µM DNA template in a buffer containing 40 mM Tris-HCl (pH 8.0), 8 mM MgCl_2_, and 2 mM spermidine and various concentrations of (a) KCl or (b) LiCl. Lane 1 shows 10-nt size marker, lane 2 shows 35-nt RNA, and lanes 3 to 7 show transcription products in the presence of 0, 10, 30, 50, and 70 mM (a) KCl or (b) LiCl. Blue and red arrows indicate the slipped and arrested transcripts, respectively.

To understand how structural stability affected the production of slipped and arrested transcripts, we further analysed transcription from template Q5, which resulted in both slipped and arrested products (Figure 2c, lane 11). We evaluated transcription in buffer with KCl concentrations ranging from 0 to 70 mM (Figure 3), because the stability of G-quadruplex strongly depends on K+ concentration.[30], [31], [32] UV measurements showed the −Δ*G* ^o^37 values of q5 increased with increasing KCl concentration (Table S4 in File S1). In the absence of KCl, the −Δ*G* ^o^37 value for q5 was −0.1 kcal mol^−1^, and slipped, but no arrested, product was produced from template Q5 (Figure 3a, lane 3). In the presence of 10 to 70 mM KCl, the arrested transcript was observed (Figure 3a, lanes 4 to 7, red arrows). The amount of slipped transcript decreased and the amount of arrested transcript increased with increasing KCl concentration. The transcription experiments were also performed in various concentrations of LiCl as G-quadruplexes are significantly destabilized in the presence of Li^+^.[33] Only slipped transcript was produced in the presence of LiCl (Figure 3b, blue arrows). Thus, the slippage was induced even when the G-quadruplex was unstable, and increasing amounts of arrest were observed as the non-canonical structure was stabilized. Only full-length transcript was produced from the liner template in the presence of KCl and LiCl (Figure S5 in File S1).

To confirm that the production of slipped and arrested transcripts was caused by formation of a G-quadruplex, transcription experiments were also performed in the presence of G-quadruplex-stabilizing ligands, 5,10,15,20-tetrakis (N-methyl-4-pridyl) porphyrine (TMPyP4), berberine, and N-methyl mesoporphyrine IX (NMN).[34], [35], [36]As we expected, more of the arrested transcript was observed for template Q5 in the presence of each ligand than without ligand in 3 mM KCl (Figure S6 in File S1). The stability of the q5 structure in the presence of each of the ligand was significantly enhanced (Table S5 in File S1). We also investigated transcription from different template DNAs (Q3, Q6, and the platelet-derived growth factor B (PDFB) sequence,[37] which contain a G-quadruplex-forming sequence). In all case, the presence of G-quadruplex forming sequences induced transcription arrest (Table S5 in File S1).

### Quantitative correlation between the stability of non-canonical structures and transcription efficiency

To quantify the effect of the non-canonical structure on the transcription, we estimated the correlation between the stability of non-canonical structures and transcription efficiency (TE). The full-length and slipped transcripts were defined as “run-off transcripts” (TE_run-off_) because the RNA polymerase travels to the end of template DNA when these products are produced (Figure 1b). The TE of transcript (%) was calculated as the proportion of the gel band intensity for each transcript from structured template DNAs to the band intensity of full-length transcript from linear template DNA (Table S6 in File S1).

The TE_run-off_ values for H1, H2, and H3 in 30 mM KCl solution were 87.3, 84.8, and 76.7%, respectively; thus, the transcription efficiency decreased with increasing hairpin stability (Table 1 and Table S6 in File S1). The TE_run-off_ values for Q1, Q2, and Q3 in 30 mM KCl solution were 98.0, 66.9, 16.2%, and those for Q4, Q5, and Q6 were 99.3, 47.5, and 4.2%, respectively (Table S6 in File S1). Moreover, the TE_run-off_ values for Q5 in 10 and 50 mM KCl solution were 68.5 and 45.0%, respectively (Table S4 in File S1). Thus, for these G-quadruplex-forming templates the transcription efficiency decreased with increasing G-quadruplex stability. In general, the TE_run-off_ should not depend on DNA sequence because the rate-determining step of transcription in multi-turnover conditions is the initiation step (that is, RNA polymerase binding to promoter region). The TE_run-off_ for templates with non-canonical structures clearly decreased depending on DNA sequence, which indicated that the pause induced by the non-canonical structures was the rate-determining step. For the hairpin-forming templates, H1, H2 and H3, the values of TE_run-off_ decreased with increasing the values of −Δ*G* ^o^37 (Figure S7a in File S1). The values of TE_run-off_ for Q1, Q2, Q4, and Q5 also decreased with increasing −Δ*G* ^o^37 (Figure S7a in File S1), but the decrement depended on sequence and topology of G-quadruplex.

We also estimated the correlation between the stability of G-quadruplex and transcription efficiency for arrested transcript (TF_arrest_). Interestingly, we found a good correlation between the stability of G-quadruplex structures in the template DNA and the TF_arrest_ (Figure S7b in File S1). The values of TF_arrest_ increased with increasing stability of the G-quadruplex. We hypothesize that the polymerase cannot unwind a stable G-quadruplex, and, therefore, the polymerase stalls. The arrested transcripts are produced when the polymerase stalls long enough to dissociate from the template.

### Correlation between the stability of non-canonical structures in the presence of polyethylene glycol and transcription efficiency

Intracellular environments are highly crowded with various biomolecules; therefore, *in vitro* studies under molecular crowding conditions provide important information on how biomolecules behave in cells.[16], [38], [39] We previously showed that the structures of DNA immobilized on a gold surface are totally different from those in dilute bulk solution due to molecular crowding of immobilized DNAs.[40] Moreover, the presence of discrete nanoscale spaces such as those inside micelles and reverse micelles induces molecular crowding and alters the stability and structures of biomolecules.[41], [42] During transcription, the template DNA duplex is taken into the active site of RNA polymerase, therefore, the environment surrounding DNA is very different from a dilute solution. We hypothesized that the polymerase essentially induces crowding conditions on the template DNA and therefore alters the stability of the structures adopted by the template. We measured the values of −Δ*G* ^o^37 for the non-canonical structures formed by the templates in the molecular crowding condition induced by 20 wt% polyethylene glycol of molecular weight 200 (PEG 200).[16], [38], [39] As expected based on previous studies,[16], [17] hairpins formed by h1, h2, and h3 were destabilized by addition of 20 wt% PEG 200. In contrast, the G-quadruplexes formed by q1, q2, q4, and q5 were stabilized, although the stabilities of q3 and q6 were too high to allow accurate determination of thermodynamic parameters. In principle, formation of DNA structures is accompanied by the formation of a hydrogen-bonding network of water surrounding the DNA surface that is highly sensitive to the water activity of the solution.[16], [17], [43] A decline in the water activity in the presence of PEG 200 disfavors formation of structures such as hairpins that require uptake of water molecules, and favors formation of structures such as G-quadruplex that are accompanied by the release of water. [16], [43]

**Figure 4:**
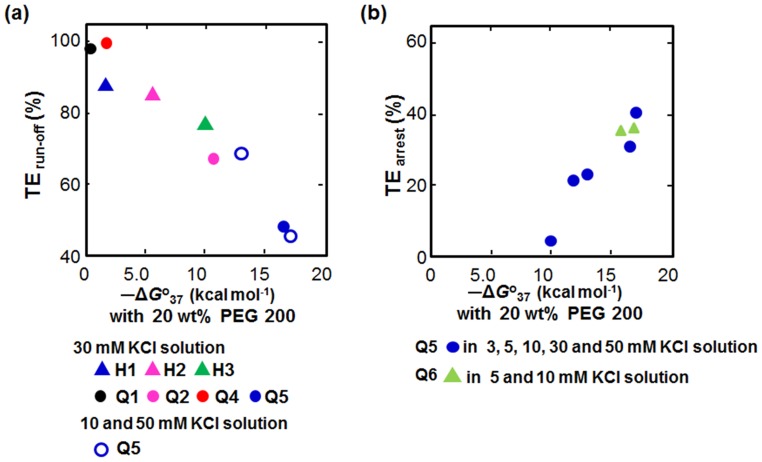
Correlation between −Δ*G* ^o^_37_ values obtained in 20 wt% PEG200 and transcription efficiency (TE). (a) TE of run-off transcripts and (b) TE of arrested transcript. Reaction mixtures contained 0.3 µM T7 polymerase and 1.5 µM DNA template in a buffer containing 40 mM Tris-HCl (pH 8.0), 8 mM MgCl_2_, and 2 mM spermidine, and reactions were incubated for 90 min at 37°C.

We estimated the correlation between the stability of non-canonical structures in the presence of 20 wt% PEG 200 and TE_run-off_ (Figure 4a). The values of TE_run-off_ decreased with increasing stability of non-canonical structures in the presence of 20 wt% PEG 200. The decrement of TE_run-off_ was not dependent on topology of non-canonical structures, although values of TE_run-off_ seemed to strongly depend on the topology of non-canonical structures in the absence of PEG. Furthermore, the amount of arrested product increased linearly with the increasing stability of non-canonical structures in the presence of 20 wt% PEG 200 (Figure 4b). Thus, the −Δ*G* ^o^37 values for the non-canonical structures in the presence of PEG are reflective of the stabilities of the non-canonical structures during transcription.

## Discussion

During transcription, pause or arrest may result from unstable hybridization between template DNA and nascent RNA or upon formation of a structure in the DNA that interrupts and perturbs elongation. The decrease of the transcription efficiency in the templates studied here results from a halt in transcription elongation at structures formed in the DNA templates. Significantly lower transcription efficiencies (as measured based on the amount of full-length transcript produced) were observed with templates able to form G-quadruplex structures relative to templates able to form hairpins (Figure 2c and Table S6 in File S1). In addition, transcription arrest was induced only by stable G-quadruplexes (Figures 2c, 3 and S6 in File S1, and Tables S4, S5, and S6 in File S1). Thus, G-quadruplex structures are more effective inhibitors of elongation than are hairpin structures, as suggested by the correlation between transcript production and the stability of structures formed by oligonucleotides of the same sequence (Figure S7 in File S1).

Our experiments in the presence of PEG 200 indicate that the conditions within the polymerase active site might be more similar to those induced by molecular crowding agents than to those in an aqueous buffer. The dielectric constant is also likely low in the region of the polymerase active site: There were several reports that the dielectric constant is very low (less than 10) inside proteins such as RNase T1.[44] We and the other groups have shown that molecular crowding conditions stabilize G-quadruplexes and destabilize hairpins.[16], [39], [45] Moreover, G-quadruplexes are stabilized when the dielectric constant is low, but duplex stabilities are unchanged or slightly destabilized. Therefore, the stabilities of the G-quadruplexes during the transcription could be higher than stabilities estimated based on our thermal melting analyses in the absence of PEG 200 and that of hairpins could be lower. Additionally, Hoogsteen base pairs are stabilized under the molecular crowding conditions[16], [39]. Thus, the PEG 200 added as the molecular crowding inducer may mimic not only molecular crowding but also intra-polymerase conditions. We observed correlations between the values of TE_run-off_ or TE_arrest_ and stabilities of non-canonical structures in the presence of 20 wt% PEG 200 (Figure 4).

A model based on the transcription elongation complex (TEC) that consists of RNA polymerase, template DNA, and nascent RNA at an abasic site suggests that transcription can be interrupted by formation of a hybrid between template DNA and nascent RNA.[8], [46], [47] We hypothesize that the mechanism of transcription interruption due to non-canonical structures differs from this model. When the polymerase encounters a non-canonical structure that is not readily denatured, the TEC complex will be destabilized. Due to the destabilization of TEC, the polymerase may slip backward or forward on the template DNA. Alternatively, the TEC may dissociate. Transcriptional arrest was induced when the stability of the structure (−Δ*G* ^o^37) in the presence of 20 wt % PEG 200 was more than 8.2 kcal mol^−1^ (the X-intercept value in Figure 4b), indicating that the polymerase is unable to melt structures more stable than 8.2 kcal mol^−1^. Patel and co-workers determined experimentally that the observed free energy changes for opening (melting) the template DNA duplex (−Δ*G*_open_) in the complex of T7 polymerase and template DNA in the promoter during transcription initiation were in the range of 7.0 to 8.0 kcal mol^−1^, different from the predicted value (∼6 kcal mol^−1^) of observed free energy changes by nearest neighbour analysis the under physiological ionic conditions.[48] Interestingly, the −Δ*G*_open_ value from the Patel's report is similar to the value we estimate based on G-quadruplex stability. Thus, the stable non-canonical structures might stall the polymerase long enough to result in its dissociation from the template. Our estimated values of non-canonical stability in the presence of 20 wt% PEG 200 are an indicator of stability necessary to result in transcription perturbation by the non-canonical structures.

Pause and a slippage is known to occur in homopolymeric A/T or C/T tracts in the template DNA[2] and can lead to production of mutant protein. It was reported that polymerase backtracking appears to pass through a paused, transcriptionally inactive intermediate state.[28], [49] The molecular basis for backtracking is not clear, but it is correlated either with mispairing[50], [51], [52] or very weak pairing of the 3′ end of the transcript and the template.[49], [53] Current models require that the RNA-DNA hybrid at the point of arrest be very weak to initiate upstream translocation.[50] During transcription elongation, structures formed by the template DNA had not been thought to induce slippage, although G-quadruplexes have been shown to induce transcription arrest and pause. [27], [46] It has also been shown that G-quadruplex formation in a promoter region of template DNA may inhibit transcription initiation. It was also suggested that a purine tract can slippage, but existing models do not account for this effect since weak hybridization at the 3′end of purine-containing transcripts are not necessary to provoke arrest.[54] We hypothesize that these purine tracts form G-quadruplex structures. Our data imply that the effects resulting from the presence of non-canonical structures depend on the energy necessary to dissociate the non-canonical structures. If the polymerase encounters a non-canonical structure at least 8–10 nt upstream of a slippery sequence, the polymerase may slip and return to the transcriptionally competent position (resulting in a long mutated transcript) or may dissociate (resulting in an arrested protein product). Hence, our findings suggest another mechanism for mutation induced by non-canonical structures.

A purine tract found in the HIV-1 sequence[55] and trinucleotide repeat (TNR) sequences[56] impact polymerase elongation. It was also shown that repeat sequence of (CG)_14_ induces large deletions in transcripts in mammalian cells.[57] The effects of these sequences are likely caused by non-canonical structures such as Z-form, G-quadruplexes, and hairpins (cruciforms). The molecular crowding conditions present inside living cells varies with the cell cycle, and non-canonical structure formation will thus be regulated by conditions such as concentration of cosolutes and cations.[38] Our observations suggest how slipped and arrested transcript formation is controlled by conditions inside cells from the viewpoint of stability of non-canonical structures at the energy level.

The regulation of transcription elongation impacts the expression of many eukaryotic and viral genes. Our results indicate that transcriptional arrest by G-quadruplex structures within the template DNA depends on the stability of the structures formed. Moreover, the transcription arrest could be induced by the addition of ligands that bind to and stabilize G-quadruplexes. These ligands have potential as drugs for cancer treatment since they inhibit gene expression via transcription regulation. Our results further our understanding of the impact of template structure on the transcription process and may guide design of transcription-regulating drugs.

## Conclusions

We evaluated the quantitative effect of the non-canonical structures (G-quadruplexes and hairpins) in template DNA on transcription. Previously, it had been thought that G-quadruplexes block elongation. Our results indicated quantitatively that transcriptional arrest, pause, and slippage can be induced by non-canonical structures and hairpins and that the effect is dependent on the structure formed and its thermal stability. We speculate that G-quadruplex structures are more effective inhibitors of full-length product formation than are hairpin structures because the intra-polymerase conditions preferentially stabilize the G-quadruplex. Thus, the perturbation of transcription fidelity due to non-canonical structures can be regulated by the stability of the structures formed in the polymerase.

## Materials and Methods

### Materials

All oligodeoxynucleotides used in this study were purified by high-performance liquid chromatography (Japan Bio Service). Single-strand concentrations of DNA oligonucleotides were calculated from the absorbance measured at 260 nm and 80°C using single-strand extinction coefficients calculated from the mononucleotide and dinucleotide data according to the nearest-neighbor approximation model.1 The absorbance was measured using a Shimadzu 1700 spectrophotometer connected to a thermoprogrammer. The concentration of T7 polymerase was determined by its absorbance at 280 nm by using ε_280_ = 1.4×10^5^ M^−1^ cm^−1^. [58]

### Circular dichroism (CD) measurements

CD measurements were made on a JASCO J-820 spectropolarimeter at 2 µM total DNA strand concentration in buffers containing 30 mM KCl, 40 mM Tris-HCl (pH 8.0 at 37°C), 8 mM MgCl_2_, and 2 mM spermidine. The spectra at 37°C were obtained by taking at least three scans from 200 to 350 nm in a cuvette with a pathlength of 0.1 cm. The temperature of the cell holder was regulated by a JASCO PTC-348 temperature controller, and the cuvette-holding chamber was flushed with a constant stream of dry N_2_ gas to avoid condensation of water on the cuvette exterior. Before the measurement, the samples were heated to 95°C, cooled at a rate of −1°C min^−1^, and incubated at 37°C for 30 min.

### Gel electrophoresis

Native gel electrophoresis was carried out on 10% nondenaturing polyacrylamide gels in buffer containing 30 mM KCl, 40 mM Tris-HCl (pH 8.0 at 37°C), 8 mM MgCl_2_, and 2 mM spermidine at 37°C. Loading buffer (1 µL of 40% glycerol and 1% blue dextran) was mixed with 2 µL of 2 µM DNA sample. Gels were stained with SYBR® Gold (PerkinElmer Life Sciences) and imaged using a fluorescent imager (FUJIFILM, FLA-5100). Before the measurement, the samples were heated to 95°C, cooled at a rate of −1°C min^−1^, and incubated at 37°C for 30 min. Unstructured DNAs of 12, 15, 25, and 30 nt were electrophoresed in parallel. When hairpin or G-quadruplex is formed, migration of the DNA should be fast relative to unstructured DNA of the same length. The migration of all template DNAs with hairpins were faster than that of the 12-nt unstructured DNA and migration of templates designed to form G-quadruplexes were faster than that of unstructured DNA of 15 nt indicating the all DNA sequences formed hairpin or G-quadruplexes.

### Fluorescence measurement of protoporphyrin IX (PPIX)

Template DNA (1 µM) were mixed with PPIX (1 µM) in buffer containing 30 mM KCl, 40 mM Tris-HCl (pH 8.0 at 37°C), 8 mM MgCl_2_, and 2 mM spermidine for 30min. Fluorescence intensity of PPIX was measured at 37°C using a spectrofluorometer (JASCO, F6500) with 400 nm excitation and 630 nm emission. Before the measurement, the samples were heated to 95°C, cooled at a rate of −1°C min^−1^, and incubated at 37°C for 30 min.

### Thermodynamic analyses

Ultraviolet (UV) absorbance was measured on a Shimadzu 1700 spectrophotometer equipped with a temperature controller. Melting curves at 260 or 295 nm were measured in buffers containing 30 mM KCl, 40 mM Tris-HCl (pH 8.0 at 37°C), 8 mM MgCl_2_, and 2 mM spermidine. Samples were heated at a rate of 0.5 or 0.1°C min^−1^; melting curves of certain oligonucleotides differed at 0.1 and 0.5°C min^−1^ (data not shown). Thermodynamic parameters (Tables 1 and S3 in File S1) were calculated using the average values obtained from curve fitting at the different DNA concentrations (2, 5, 10, and 20 µM). Before the measurements, the DNA samples were heated to 95°C, cooled to 0°C at a rate of −0.5°C min^−1^, and incubated at 0°C for 30 min.

### Transcription assays

Unless otherwise noted, transcription reactions were carried out at 37°C in a total volume of 20 µl. T7 RNA polymerase was present at 0.3 µM, with DNA template at 1.5 µM. After incubating at 37°C for 10 min, NTPs were added to a final concentration of 1 mM each to initiate the reaction. The final reaction buffer contained 30 mM KCl, 40 mM Tris-HCl (pH 8.0 at 37°C), 8 mM MgCl_2_, 2 mM spermidine, and 5 mM DTT. Reactions were quenched after incubation at the time indicated by addition of DNase I. After incubation for 10 min, a 20-fold excess volume of transcription stop solution (80 wt% formamide, 10 mM Na_2_EDTA, and 0.1% blue dextran) was added. The samples were then heated to 90°C for 5 min, cooled rapidly, and then loaded onto a 10% polyacrylamide, 7 M urea gel run at 60°C. After electrophoresis, the gels were stained by SYBR® Gold (PerkinElmer Life Sciences), and levels were quantified with a fluorescent imager (FUJIFILM, FLA-5100). The transcription efficiency of product RNA (%) was calculated as the proportion of the gel band intensity for product RNA of each template DNAs to full-length product of linear DNA.

To confirm that transcription from Linear was not affect by conditions, we examined the transcript RNA produced by template DNA with (CT)_7_ (sequence name: CT). CT was designed to have a typical slippage sequence of (CT)_7_ at a site 35 bases downstream from the T7 promoter region. From the CT template, the intensity of the full-length transcript relative to the full-length transcript from Linear was very low. Moreover, transcripts migrating slower and faster relative to full-length transcript were produced from CT template. The Linear template produced mainly full-length transcript and the production amount full-length transcript from Linear was very high suggesting no perturbation of transcript fidelity. In addition, the intensities in the region of transcripts migrating slower (area A in Figure S3 in File S1) and faster (area B in Figure S3 in File S1) than full-length relative to the intensity of full-length transcript were very low from the Linear template (less than 30% relative to CT). These measurements indicate that little slippage occurs on the Linear template.

## Supporting Information

File S1 (PDF)
