# Supplementary material for: New Insights into Transcription Fidelity: Thermal Stability of Non-Canonical Structures in Template DNA Regulates Transcriptional Arrest, Pause, and Slippage
Source: PLoS One. 2014 Mar 3;9(3):e90580. doi: 10.1371/journal.pone.0090580 (PMC3940900; doi:10.1371/journal.pone.0090580)
Supplement: File S1 — (PDF) [file pone.0090580.s001.pdf]

Supplementary Information

New Insights into Transcription Fidelity: Thermal Stability of Non-canonical Structures in Template DNA Regulates Transcription Arrest, Pause, and Slippage

Hisae Tateishi-Karimata,[1] Noburu Isono[2] and Naoki Sugimoto[1,2] *

[1]Frontier Institute for Biomolecular Engineering Research (FIBER), Konan University, 7-1-20 Minatojimaminamimachi, Kobe, 650-0047, Japan

[2]Faculty of Frontiers of Innovative Research in Science and Technology (FIRST), Konan University, 7-1-20 Minatojimaminamimachi, Kobe, 650-0047, Japan

Table S1. Sequences of the DNA templates used in this study

Abbreviation	Sequence[a,b]
Anti-T7 promoter	5´ – GATCACTAATACGACTCACTATAGG – 3´
Linear	5´ – GCCGTTTCGTAGTATTTGGGTTGTAACTATCGAGGTTCTACACTCA<br>CTCTCTCTACTTCATATCATCTCCTATAGTGAGTCGTATTAGTGATC – 3´
H 1	5´ – GCCGTTTCGTAGTATTTCTATCC***GGACTTCGGTCC***TTGTAACTATC<br>GGGTGTGTAGTTCGTGTCATCTCCTATAGTGAGTCGTATTAGTGATC – 3´
H 2	5´ – GCCGTTTCGTAGT***CTATCGGACTTCGGTCCGATAG***TTGTAACTATC<br>GGGTGTGTAGTTCGTGTCATCTCCTATAGTGAGTCGTATTAGTGATC – 3´
H 3	5´ – GCCGTTTCG***CCAACTATCGGACTTCGGTCCGATAGTTGG***AACTATC<br>GGGTGTGTAGTTCGTGTCATCTCCTATAGTGAGTCGTATTAGTGATC – 3´
Q 1	5´ – GCCGTTTCGTAGTATTTC***GGTTAGGTTAGGTTAGG***TTGTAACTATC<br>GGGTGTGTAGTTCGTGTCATCTCCTATAGTGAGTCGTATTAGTGATC – 3´
Q 2	5´ – GCCGTTTCGTAGTA***GGGTTAGGGTTAGGGTTAGGG***TTGTAACTATC<br>GGGTGTGTAGTTCGTGTCATCTCCTATAGTGAGTCGTATTAGTGATC – 3´
Q 3	5´ – GCCGTTTCGT***GGGGTTAGGGGTTAGGGGTTAGGGG***TTGTAACTATC<br>GGGTGTGTAGTTCGTGTCATCTCCTATAGTGAGTCGTATTAGTGATC – 3´
Q 4	5´ – GCCGTTTCGTAGTATTTCTA***GGTTGGTGTGGTTGG***TTGTAACTATC<br>GGGTGTGTAGTTCGTGTCATCTCCTATAGTGAGTCGTATTAGTGATC – 3´
Q 5	5´ – GCCGTTTCGTAGTATT***GGGTTGGGTGTGGGTTGGG***TTGTAACTATC<br>GGGTGTGTAGTTCGTGTCATCTCCTATAGTGAGTCGTATTAGTGATC – 3´
Q 6	5´ – GCCGTTTCGTAG***GGGGTTGGGGTGTGGGGTTGGGG***TTGTAACTATC<br>GGGTGTGTAGTTCGTGTCATCTCCTATAGTGAGTCGTATTAGTGATC – 3´

[a] Red: Sequence of the non-canonical structure.

[b] Underline: Sequence of the T7 promoter.

Table S2. Sequences of oligonucleotides containing non-canonical structure regions in the template DNA used for thermodynamic analyses

Abbreviation	Sequence
linear	5′– ATTTGGGTTGTAACTATCGAGG–3′
h 1	5′–GGACTTCGGTCC–3′
h 2	5′– CTATCGGACTTCGGTCCGATAG–3′
h 3	5′– CCAACTATCGGACTTCGGTCCGATAGTTGG–3′
q 1	5′– GGTTAGGTTAGGTTAGG–3′
q 2	5′– GGGTTAGGGTTAGGGTTAGGG–3′
q 3	5′– GGGGTTAGGGGTTAGGGGTTAGGGG–3′
q 4	5′– GGTTGGTGTGGTTGG–3′
q 5	5′– GGGTTGGGTGTGGGTTGGG–3′
q 6	5′– GGGGTTGGGGTGTGGGGTTGGGG–3′

Table S3. Thermodynamic parameters for hairpin and G-quadruplex formation[a]

	ΔH° (kcal mol^{-1})	$T\Delta S^\circ$ (kcal mol^{-1})
h 1	−38.6 ± 1.9	−35.9 ± 2.0
h 2	−57.2 ± 3.5	−50.0 ± 3.2
h 3	−104 ± 1	−91.3 ± 1.2
q 1	−36.1 ± 1.0	−36.0 ± 0.5
q 2	−48.8 ± 1.3	−45.1 ± 1.2
q 3	n.d.[b]	n.d.[b]
q 4	−41.1 ± 1.8	−39.7 ± 1.7
q 5	−113 ± 2	−98.7 ± 1.7
q 6	n.d.[b]	n.d.[b]

[a]All experiments were carried out in a buffer containing 30 mM KCl, 40 mM Tris-HCl (pH 8.0), 8 mM $MgCl_2$, and 2 mM spermidine. Thermodynamic parameters were evaluated using the average values obtained from curve fitting at the different DNA concentrations.

[b]The thermodynamic parameters could not be determined because of very high stability.

Table S4. The stability of q5 at various concentrations of KCl [a]

KCl concentration	T_m (˚C) [b]	$-\Delta G^{o}_{37}$ (kcal mol^{-1})	Run-off transcripts (%) ($TE_{run\text{-}off}$)	Arrest transcript (%)[c] (TE_{arrest})
0 mM	35.3	–0.1±0.4	92.5	none
10 mM	74.7	11.9±0.5	68.5	23.4
50 mM	86.2	16.7±0.2	45.0	41.6
70 mM	87.5	n.d.[d]	38.0	42.2

[a]All experiments were carried out in a buffer containing 40 mM Tris-HCl (pH 8.0), 8 mM $MgCl_2$, 2 mM spermidine, and 0, 10, 50, or 70 mM KCl. Thermodynamic parameters were evaluated using the average values obtained from curve fitting at the different DNA concentrations.

[b] The melting temperature was determined at a strand concentration of 2 μM.

[c]Arrest was defined as more than 4% production of arrested product RNA. The error value of transcription efficiency for each sample was within 6%.

[d]The thermodynamic parameters could not be determined because of very high stability.

Table S5. The stability of G-quaruplexes under various conditions[a]

Sequence	Condition	$-\Delta G^{o}_{37}$ (kcal mol^{-1})	Arrested transcript (%)[b] (TE_{arrest})
Q5	3 mM KCl<br>+ 2 μM TMPyP4	9.6±0.9	18.1
Q5	3 mM KCl<br>+ 2 μM berberine	11.6±0.4	23.2
Q5	3 mM KCl<br>+ 2 μM NMN	13.0±1.1	33.5
Q5	3 mM KCl	7.9±0.4	4.1
Q5	5 mM KCl	10.2±0.5	21.0
Q3	5 mM KCl	11.5±0.4	26.0
Q3	10 mM KCl	13.2±0.4	34.1
Q6	5 mM KCl	14.0±0.6	35.6
Q6	10 mM KCl	14.6±0.7	35.7
PDGFB	3 mM KCl	14.9±0.5	39.7

[a] All experiments were carried out in a buffer containing 40 mM Tris-HCl (pH 8.0), 8 mM $MgCl_2$, 2 mM spermidine, and 3, 5, or 10 mM KCl. Thermodynamic parameters were evaluated using the average values obtained from curve fitting at the different DNA concentrations.

[b] Arrest was defined as more than 4% production of arrested product RNA. The error value of transcription efficiency for each sample was within 6%.

Table S6 . Transcription efficiency[a]

	Transcription efficiency (%)	
	Run-off transcripts ($TE_{run\text{-}off}$)	arrested transcript (TE_{arrest})
Linear	100	
H1	87.3	
H2	84.8	
H3	76.7	
Q1	98.0	
Q2	66.9	
Q3	16.2	48.9
Q4	99.3	
Q5	47.5	31.9
Q6	4.2	58.1

[a]The transcription efficiency of product RNA (%) was calculated as the proportion of the gel band intensity for product RNA of each template DNA to full-length product of linear template DNA. The error value of transcription efficiency for each sample was within 8%.

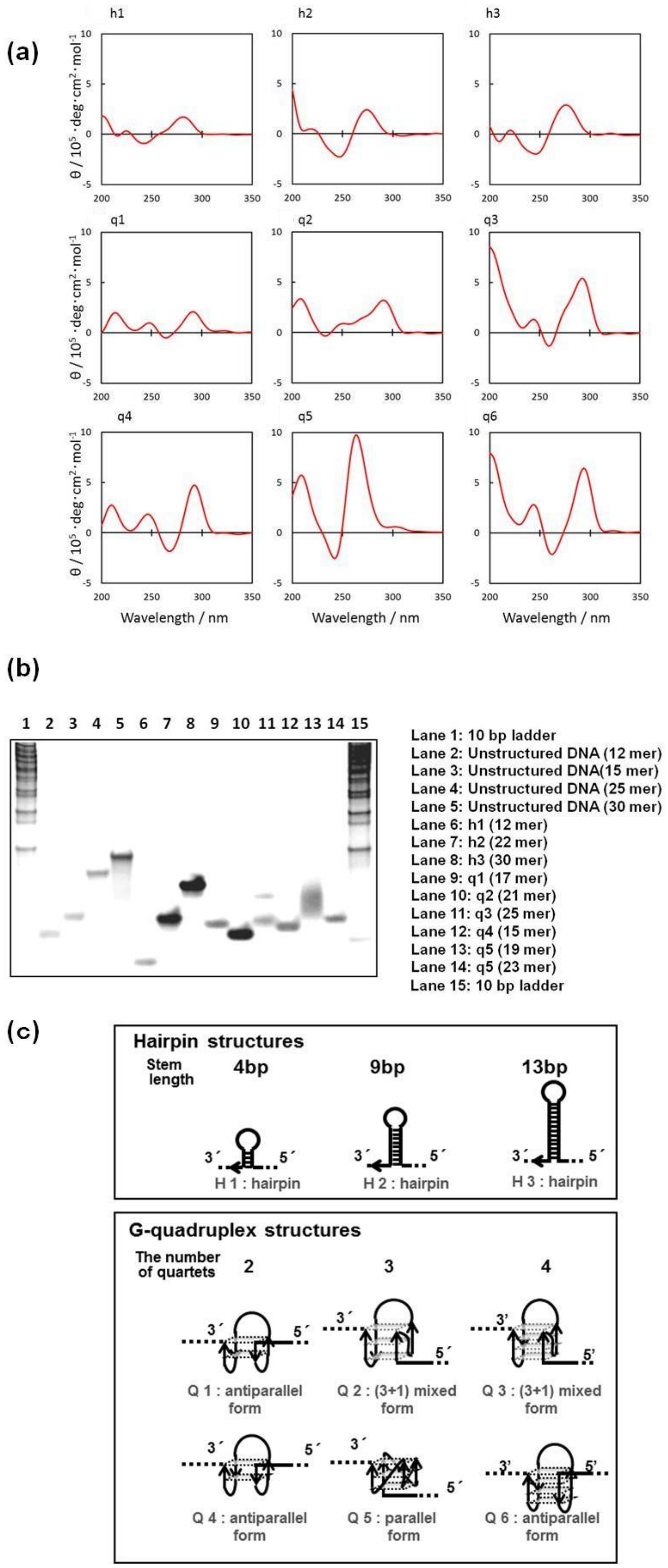

Figure S1. (a) CD spectra for 5 μM h1, h2, h3, q1, q2, q3, q4, q5, and q6 in a buffer containing 30 mM KCl, 40 mM Tris-HCl, 8 mM $MgCl_2$, and 2 mM spermidine at 37 °C. (b) Native gel electrophoresis of DNA oligonucleotides. Gels were run at 150 V and at 37 °C for 120 min in a buffer containing 30 mM KCl, 40 mM Tris-HCl (pH 8.0), 8 mM $MgCl_2$, and 2 mM spermidine. The migration of all template DNAs with hairpin were faster than that of unstructured DNA of 12 nt, and migration of templates that form G-quadruplexes were faster than unstructured DNA of 15 nt. This indicates that these templates fold into more compact structures than the unstructured DNA. (c) Schematic structures of the template DNAs revealed by CD spectra and gel analysis.

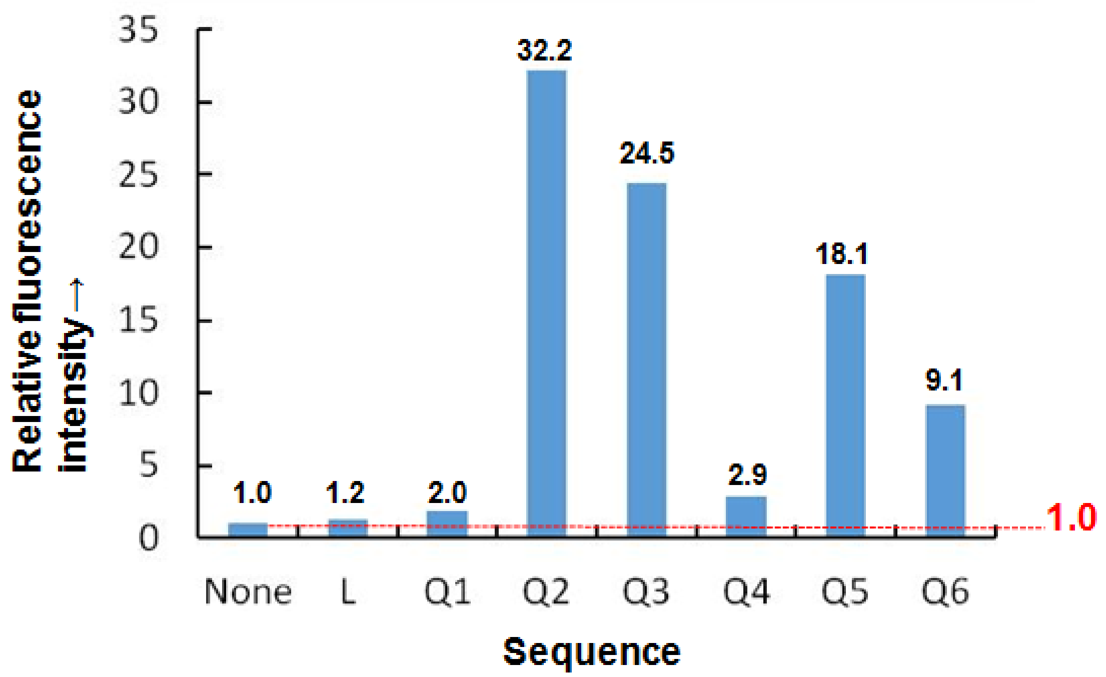

Figure S2. Relative fluorescence intensities of protoporphyrin (PPIX) with template DNAs were measured at 37 °C in a buffer containing 30 mM KCl, 40 mM Tris-HCl (pH 8.0), 8 mM $MgCl_2$, and 2 mM spermidine. The relative fluorescence intensity at 610 nm was obtained by dividing fluorescence intensity in the presence of template DNA by that in the absence of template DNA. The relative fluorescence intensities for Q1 to Q6 were more than 2 indicating the G-quadruplex formation; the relative fluorescence intensity for Linear was only 1.2.

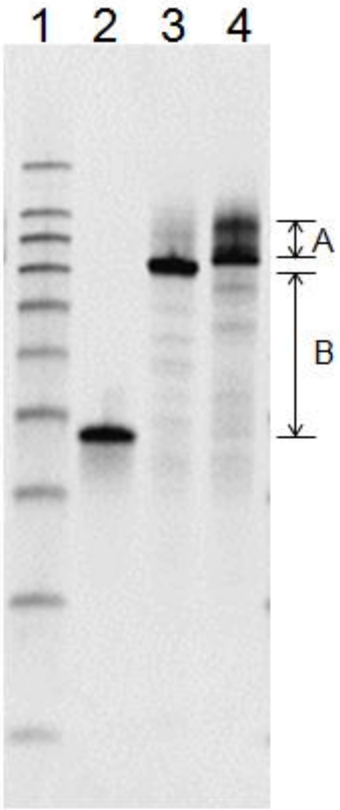

Figure S3. Denaturing gel electrophoresis of transcripts obtained from transcription of templates Linear and CT. Reaction mixtures were incubated at 37 °C for 90 min and contained 0.3 μM T7 polymerase and 1.5 μM DNA template with 1.5 μM complementary strand in a buffer containing 30 mM KCl, 40 mM Tris-HCl (pH 8.0), 8 mM $MgCl_2$, and 2 mM spermidine. Lane 1, size marker; lane 2, 35-nt RNA; and lanes 3 and 4, transcription products for Linear and CT, respectively. Transcripts in areas A and B are produced by slippage.

(a)

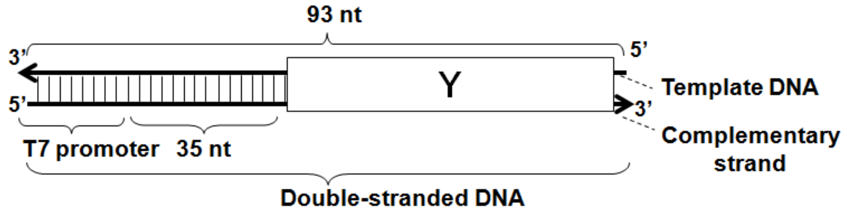

(b)

Sequence name	Sequence of Y region
dsLinear:	5'-GCCGTTTCGTAGTATTTGGGTTGTAACTATCGAGG-3'<br>3'-CGGCAAAGCATCATAAACCCAACATTGATAGCTCC-5'
dsH1 :	5'-GCCGTTTCGTAGTATTTCTATCC**GGACTTCGGTCC**-3'<br>3'-CGGCAAAGCATCATAAAGATAGGCCTGAAGCCAGG-5'
dsH2 :	5'-GCCGTTTCGTAGT**CTATCGGACTTCGGTCCGATAG**-3'<br>3'-CGGCAAAGCATCAGATAGCCAGAAGCCAGGCTATC-5'
dsH2 :	5'-GCCGT**CCAACTATCGGACTTCGGTCCGATAGTTGG**-3'<br>3'-CGGCAGGTTGATAGCCTGAAGCCAGGCTATCAACC-5'
dsQ1 :	5'-GCCGTTTCGTAGTATTTC**GGTTAGGTTAGGTTAGG**-3'<br>3'-CGGCAGGTTGACATAAAGCCAATCCAATCCAATCC-5'
dsQ2 :	5'-GCCGTTTCGTAGTA**GGGTTAGGGTTAGGGTTAGGG**-3'<br>3'-CGGCAGGTTGACATCCCAATCCCAATCCCAATCCC-5'
dsQ3 :	5'-GCCGTTTCGT**GGGGTTAGGGGTTAGGGGTTAGGGG**-3'<br>3'-CGGCAAAGCACCCCAATCCCCAATCCCCAATCCCC-5'
dsQ4 :	5'-GCCGTTTCGTAGTATTTCTA**GGTTGGTGTGGTTGG**-3'<br>3'-CGGCAAAGCACCCCAAAGATCCAACCACACCAACC-5'
dsQ5 :	5'-GCCGTTTCGTAGTATT**GGGTTGGGTGTGGGTTGGG**-3'<br>3'-CGGCAAAGCACCCCAACCCAACCCACACCCAACCC-5'
dsQ6 :	5'-GCCGTTTCGTAG**GGGGTTGGGGTGTGGGGTTGGGG**-3'<br>3'-CGGCAAAGCATCCCCCAACCCCACACCCCAACCCC-5'

(c)

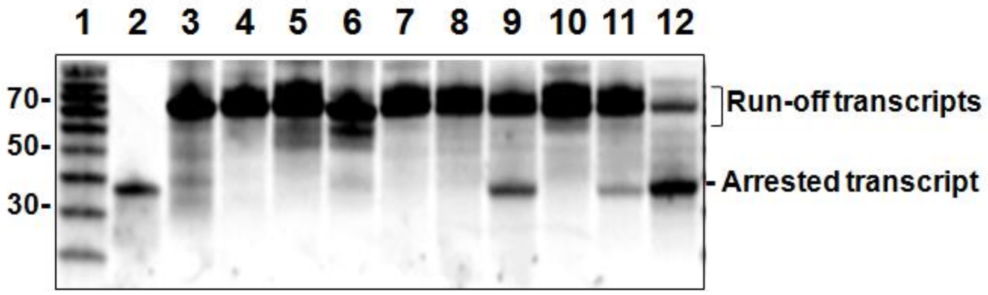

Figure S4. (a) Illustration of the template DNA and its complementary strand. The region denoted by the box labeled Y forms a duplex or a non-canonical structure. (b) Sequence names and sequences of Y regions. Sequences expected to form non-canonical structures are indicated in bold. (c) Denaturing gel electrophoresis of transcripts obtained from transcription of templates in the presence of complementary strands. Reaction mixtures were incubated at 37 °C for 90 min and contained 0.3 μM T7 polymerase and 1.5 μM DNA template with 1.5 μM complementary strand in a buffer containing 30 mM KCl, 40 mM Tris-HCl (pH 8.0), 8 mM $MgCl_2$, and 2 mM spermidine. Lane 1, size marker; lane 2, 35-nt RNA; and lanes 3 to 12, transcription products for dsLinear, dsH1, dsH2, dsH3, dsQ1, dsQ2, dsQ3, dsQ4, dsQ5, and dsQ6.

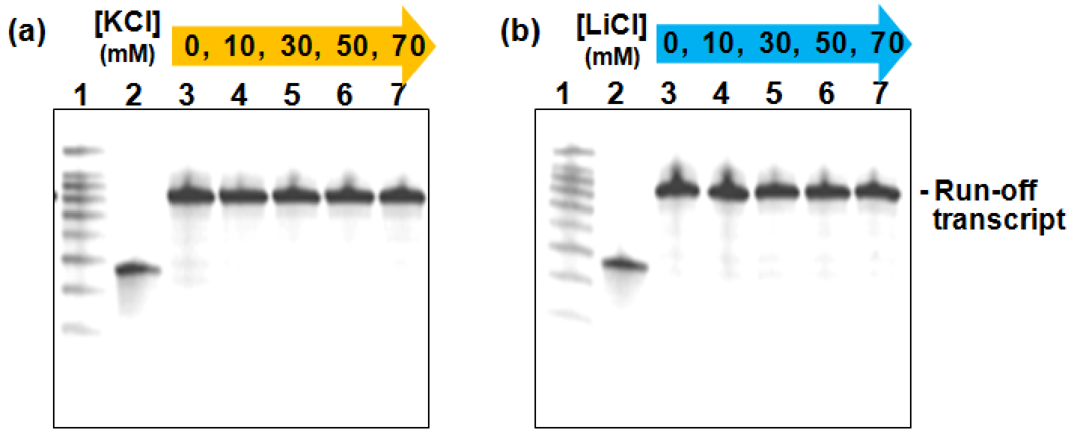

Figure S5. Denaturing gel electrophoresis of transcripts of 90-min transcription reactions at 37°C using linear template DNA. Reaction mixtures contained 0.3 µM T7 polymerase and 1.5 µM DNA template in a buffer containing 40 mM Tris-HCl (pH 8.0), 8 mM $MgCl_2$, and 2 mM spermidine and various concentrations of (a) KCl or (b) LiCl. Lane 1 shows 10-nt size marker, lane 2 shows 35-nt RNA; and lanes 3 to 7 show transcription product in the presence of 0, 10, 30, 50, and 70 mM (a) KCl or (b) LiCl.

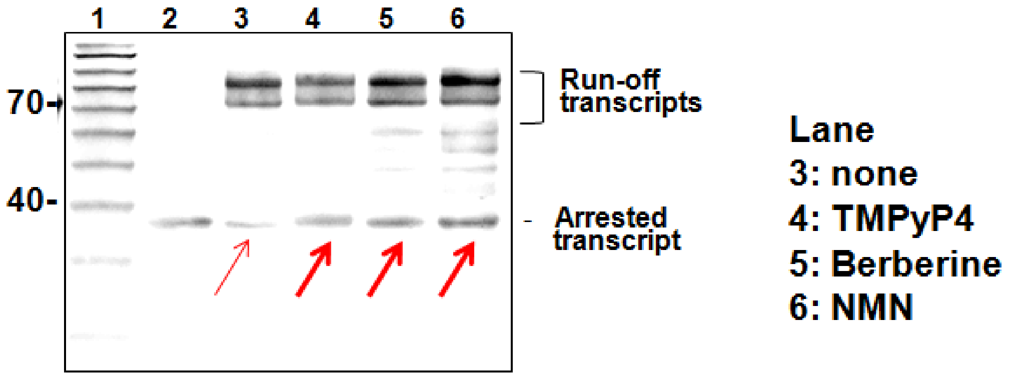

Figure S6. Denaturing gel electrophoresis of transcripts from the Q5 template DNA. Reaction mixtures were incubated for 90 min at 37 °C and contained 0.3 μM T7 polymerase and 1.5 μM DNA template in a buffer containing 40 mM Tris-HCl (pH 8.0), 8 mM $MgCl_2$, 2 mM spermidine, and 3 mM KCl in the absence (lane 3) or presence of TMPyP4 (lane 4), berberine (lane 5) or NMN (lane 6). Lane 1 shows 10-nt size marker; lane 2 shows 35-nt RNA.

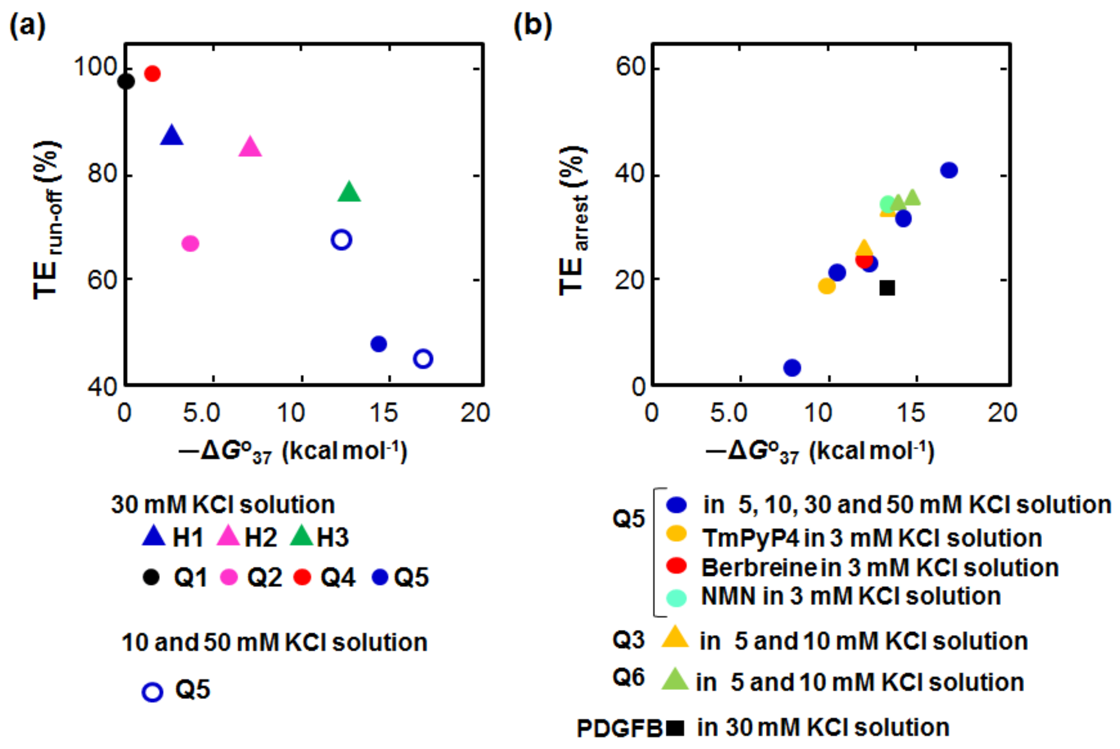

Figure S7. Correlation between $–\Delta G^{o}_{37}$ values for non-canonical structures in the template DNA and transcription efficiency (TE). (a) TE of run-off transcripts and (b) TE of arrested transcript**.** Reaction mixtures were incubated for 90 min at 37 °C and contained 0.3 μM T7 polymerase and 1.5 μM DNA template in a buffer containing 40 mM Tris-HCl (pH 8.0), 8 mM $MgCl_2$, 2 mM spermidine, and 3 mM KCl
